# Evaluation of an online systematic review escape room for early career clinicians and doctoral students

**DOI:** 10.5195/jmla.2025.2167

**Published:** 2025-10-23

**Authors:** Paul Cannon, Tracey McKee

**Affiliations:** 1 paul.cannon@glasgow.ac.uk, University of Glasgow, Glasgow, Scotland; 2 Tracey.McKee@nhs.scot, NHS Greater Glasgow & Clyde, Glasgow City, Glasgow, Glasgow, Scotland

**Keywords:** Escape Rooms, Active learning, Systematic reviews, Search skills, Research students

## Abstract

**Background:**

Systematic reviews are increasingly appearing in doctoral theses and being supported by librarians. There is, however, evidence that students struggle to undertake systematic reviews.

**Case Presentation:**

We sought to understand the perspectives of, and confidence utilising systematic review search methods following an online escape room teaching intervention as part of our in-person orientation session for Doctorate in Clinical Psychology trainees. Following the session, trainees were invited to participate in an online survey to which we received a 90% response rate (n=35). The escape room was enjoyed by most trainees with many using the words “fun” and “engaging” to describe the intervention, this despite more participants finding the escape room difficult. The average scores for confidence in utilising search syntax were positive, but there was a wide range of scores. Many of the comments that trainees made centred on time pressure to escape. We believe that allowing the trainees more time would increase their enjoyment of the game and aid their learning.

**Conclusion:**

Our systematic review escape room demonstrates that key methodological concepts and search skills can be taught in an active, fun, and engaging way that helps introduce and scaffold learning for later in-depth teaching.

## BACKGROUND

Systematic reviews (SRs) are an emerging role for academic libraries [1], resulting in many implementing dedicated SR services [2–4]. In addition, SRs have become an expected part of doctoral study [5], with some authors calling for SR methodologies to be a mandatory component of doctoral training [6]. Indeed, in the context of this case report, SRs appear in all theses.

Undertaking any type of literature review can be a novel task for many graduate students, who often encounter difficulties comprehending different review methodologies, data management requirements, and writing methods [7]. It is perhaps no surprise that SRs can be “a daunting task” for PhD students [5, p.535].

To support these students, many academic health libraries have implemented dedicated SR services for collaboration and training (for example McKeown and Ross-White [2], Yang et al. [3], and Demetres et al. [4]). Where teaching practices of librarians have been explored, didactic teaching methods dominate, with far fewer librarians engaging in active learning techniques such as collaborative group work, think-pair-share, and gamification of learning [8]. This lack of active learning utilisation is despite a growing body of literature demonstrating that students learn more when actively engaged within the classroom [9].

As a way of engaging students, escape rooms have been used in many disciplines [10–12]. Within libraries, escape rooms have also been used for library orientations and literature searching [13], fact checking and fake news [14], and searching PubMed [15]. These library-focused escape rooms were not sufficiently evaluated to show increased learning or knowledge retention but do show intended learning outcomes were met and that participants enjoyed playing them.

Whilst there is a paucity of literature on clinical psychology within health librarianship, clinical psychology meta-analyses show better, but similarly low levels of search strategy reporting as other health disciplines [16]. In an effort to improve trainee confidence in searching and reporting for SRs, we have embraced active learning, utilising approaches that enable trainees to apply knowledge from teaching [17].

In this study, we investigated whether an additional active learning intervention in the form of an online escape room during our orientation with trainees in the Doctorate in Clinical Psychology (DClinPsy) programme would be an effective way to introduce SR methods and search skills earlier in the curriculum and help scaffold future learning.

## CASE PRESENTATION

We are two librarians working in a research-intensive university and a health service within the same geographic region. We support DClinPsy trainees on a programme that is collaboratively funded by both organisations. The trainees are also both health service workers and enrolled students during the three-year programme. Whilst the two organisations are separate, with access to both libraries, trainees often conflate our services. We therefore began joint orientation sessions to help define our services and direct trainees to the appropriate support for academic and clinical enquiries. Further conflation existed with a requirement for trainees to undertake a SR for their academic theses, which is linked to a research project undertaken in a clinical setting. Our collaboration, therefore, quickly extended to co-design and delivery of SR teaching.

We have three teaching interventions embedded within the DClinPsy curriculum that enable us to scaffold learning across the first two years of study. These are all timetabled and mandatory to attend. Our interventions start in the first weeks of year one with an hour in-person orientation session. This is followed by a 1.5-hour online lecture at the end of the first semester where SR methodologies and search methods are explained. This lecture aligns with the submission of their research project proposals, which influences their SR topic. The outline of their SR is due at the end of semester two in year one.

Our final timetabled session is a full day online SR search and reporting workshop midway through the first semester of year two. By the end of the workshop, we aim for trainees to have a first draft of a search strategy on one database consisting of subject headings and text words, with appropriate utilisation of syntax and search fields. This workshop marks the start of their SR, with trainees expected to write their reviews over the next year, submitting them as part of their theses mid-way through their third and final year.

Despite our teaching interventions, optional appointments, and online support resources, we encounter some trainees with low confidence towards their SRs. Specifically, we have noticed that many questions could be answered by handbooks (for example, from The Cochrane Collaboration [18] and JBI Manual for Evidence Synthesis [19]), that trainees struggled to differentiate systematic and scoping review research questions, and with nesting, proximity searching, and wildcards.

To further support trainees in these areas, we decided to experiment by utilising an escape room in our year one orientation session. To the best of our knowledge, our escape room is the first to focus on methodological issues and search skills in SRs. Our escape room can be downloaded and played from https://osf.io/jwf6t/.

### Escape room development

Our online escape room was created using Microsoft OneNote following an instructional case study [20] and video tutorial [21]. The short video tutorial shows how to create pages within OneNote for each puzzle and password protect answer pages. All our puzzles are in the form of multiple-choice questions; the correct answer is the password to unlock the next section of the escape room. Each correctly answered section reveals a character which, when unscrambled, reveals the code to complete the escape room. The questions focus on methodological guidance, Boolean logic, and search syntax. The escape room navigation and question development are discussed in Appendix A.

### Using the escape room in class

For our orientation in academic year 2024/25, the trainees were randomly divided into two sets of 19 and 20 people by the programme administration team due to room capacity. When entering the room, trainees were free to choose their seats around one of four active learning tables. This meant that there were eight groups of approximately five people across both repeated orientations.

Each session lasted one hour. The first 20 minutes of the orientation included a presentation on library services and group information retrieval exercises from our library catalogue. We then introduced the escape room and navigation for five minutes, gave the trainees 15 minutes to complete the escape room, and spent the final ten minutes providing context to the questions and explaining the answers. We allowed ourselves ten minutes for a welcome and closing of the orientation and questions from the trainees. During the escape room exercise we checked in with each table, answering any technical questions and providing prompts as required.

When closing the session, we introduced our study and invited trainees to undertake an anonymous online survey. A further email with details of the survey was sent to trainees immediately after the session by the DClinPsy admin team on our behalf, with a reminder sent one week later. The survey closed after four weeks.

### Survey development

Our survey was modified from Offord et al. [20] and PRESS guidelines [22] to be suitable to the trainees and search methods. The survey employed five-point Likert scales and free-text open-ended responses. Quantitative questions were analysed using simple descriptive statistics, with free-text questions using the constant comparison method [23], allowing us to quickly generate broad codes and themes based on similarities and differences in the data. As an example of our coding, one response to whether there were parts of the game participants found difficult was, “The time pressure of the activity and reaching group consensus on some questions was difficult. However it was helpful to discuss and reach concensus (sic) while building our understanding”. This response was coded as “Teamworking” and “Time pressure”, and added to the theme, “Game experience”. The raw survey data and our coding structure are available on the Open Science Framework [24].

We received a 90% response rate (35 from 39 potential participants). We believe the high response rate corresponds with our valued input and trainee enjoyment of the escape room. The survey was also short, taking a mean average of 3 minutes 36 seconds to complete, and all questions were optional.

## RESULTS

### Escape room gameplay

**Figure 1:**
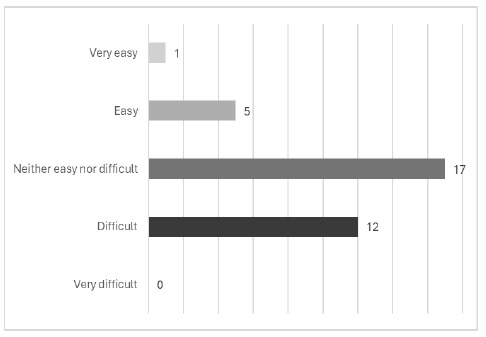
Responses to the question, “How easy or difficult did you find the game?”

The initial questions in our survey used a five-point Likert scale to assess whether the trainees enjoyed playing the escape room and whether they found the game easy or difficult. The options ranged from ‘Really enjoyed it' to ‘Did not enjoy it at all' and ‘Very easy' to ‘Very difficult', respectively. The escape room was near universally enjoyed by all participants with 60% (n=21) ‘really enjoying' and 34% (n=12) ‘enjoying' it. 6% (n=2) felt ‘neutral'. Trainees' enjoyment of the intervention came despite a larger percentage finding the escape room ‘difficult' or ‘neither easy nor difficult' (Figure 1).

The reasons for people finding the escape room difficult were elicited in responses to the question, “Were there particular parts of the game that you found difficult?”. Time pressure was coded on a quarter of responses (n=9/33). A similar number (n=6) mentioned difficulties with search skills, particularly with questions on wildcards and proximity searching. A further five comments were coded with team working. These responses centred on group organisation and lack of assigned roles, resulting in individuals working at a different pace, “Doing it in a big group meant we all couldn't read at the same pace, so ended up not all being on the same wavelength”. Several comments were coded to cognitive load, which may be partly related to time pressure and lack of group working when compared to our pilot of the escape room with the previous 2023/24 cohort. One person related cognitive load to their neurodivergence. We reflect on these factors in the discussion section. Five responses noted no difficulties with the escape room, but one person said “all of it” was difficult.

### Development of search skills

In response to, “What did you learn from the game?”, participants answers were coded to syntax (n=6/35), Boolean logic (n=4), and general comments on search skills (n=9). Most responses were assigned a broad methodology theme (n=18), which included codes for the general steps in a SR (n=14), with five of these specifically mentioning, and coded to, guidelines and handbooks. Five responses were also coded under previous SR experience, which highlights the range of experience within this cohort.

**Figure 2:**
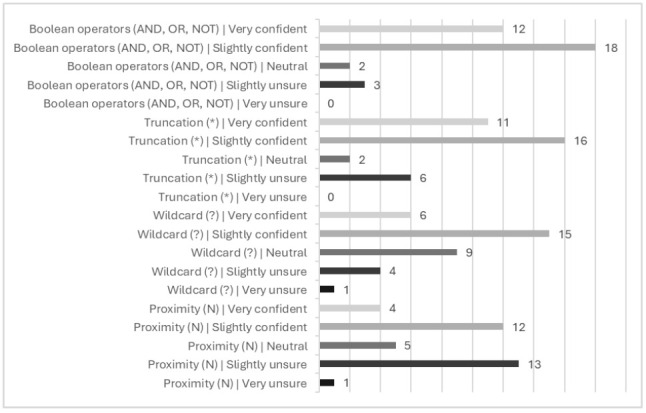
Responses on a five-point Likert scale to the question, “Following the escape room, how confident do you feel utilising the following techniques as part of a systematic review search strategy?”

Trainees were then asked how confident they felt utilising search techniques (figure 2). Across all four measured search techniques, more people were slightly or very confident utilising these search methods than very or slightly unsure. However, the data shows a wide variation in confidence levels indicating that some trainees require further support with search techniques (table 1). We reflect on these responses in the discussion.

**Table 1:** Range and average scores for responses to the question, “Following the escape room, how confident do you feel utilising the following techniques as part of a systematic review search strategy?” (where 1=very unsure, 2=slightly unsure, 3=neutral, 4=slightly confident, 5=very confident)

Search technique	Minimum	Maximum	Mean	Count
**Boolean operators (AND, OR, NOT)**	2	5	4.11	35
Truncation (*)	2	5	3.91	35
**Wildcard (?)**	1	5	3.60	35
**Proximity (N)**	1	5	3.14	35

### Overall perceptions of the escape room

We asked participants, “Would you recommend this game to other students undertaking a systematic review?”. Of the 35 responses, 32 replied “yes” and 3 people “maybe”. When asked to explain their answer, of the 29 free-text responses, 16 were coded as fun and ten as engaging, with these exact words often used. Nine responses were coded with bite-sized learning, with participants observing the escape room as, “a nice way to explain the knowledge concisely with easy to understand examples” and that, “It really helped get me thinking about the different aspects of a SR”. Two of these trainees bookmarked the escape room to review later. Two people mentioned positive aspects of team working through knowledge sharing, and that it “encouraged conversation”. Of the three people that said they would “maybe” recommend the game, one person noted that it was fun but that they did not learn anything new, and the other person said, noting their neurodivergence, that they required more time and repeated self-directed attempts for the learning to “stick”. The third person left the open response blank.

We concluded by asking, “Is there anything else you would like to tell us about the game?”. From the 23 responses most noted the fun (n=8) aspect of the escape room, praising its novelty (n=8). We were also thanked for trying something different (n=6), “It was a great way to itroduce (sic) these skills in an accessible way”. Two people noted teamwork, one enjoying the “positive bonding elements”, and the other offering that if they were able to play the escape room alone, “I think [it] would be more useful, and could be longer or more complex!”.

## DISCUSSION

Overall, our escape room can be considered a success by the measures of enjoyment, engagement, and beginning to scaffold learning. With many doctoral theses requiring SRs, and more libraries supporting SRs, escape rooms may be “a helpful engaging way of learning more about a dry topic”, as one of our participants noted. There are, however, some areas where refinement of how the escape room is introduced and managed would benefit future cohorts and libraries seeking to develop similar interventions.

### Pressure to escape rather than develop knowledge

One of the most common codes in the analysis was time pressure. The orientation timetable necessitates a short time for the escape room. Trainees also noted positive and negative aspects of the time limit; some feeling that it improved engagement and gameplay, whilst others felt pressure to escape rather than reflect on their learning. We based the 15-minute escape time on our experience with the previous cohort, and whilst all groups escaped within 15-minutes, many responses on the difficulty with the game stemmed from time pressure. One participant commented, “Obviously a time crunch is part of the game but it was a little hard to process some of the instructions in such a short pace of time. Maybe make it 20?”. Other participants noted there was not sufficient time to work collegiately within their groups. We observed that there was less team working within this cohort compared to the last. To what extent this was due to the time limit or group dynamics is difficult to tell. Nonetheless, we feel 15-minutes was appropriate for escaping, but greater team working and learning could be achieved with an additional 5-10 minutes to escape. We will add this time into future orientations.

As noted, group dynamics were different between cohorts of trainees. The 2023/24 cohort were more self-organising – one person read the escape room instructions and questions, and the other team members worked in groups of two, sharing laptops, to investigate each possible answer to the questions. These trainees discussed their findings as they progressed, helping the groups share learning, collaboratively decide upon the answer, and escape quicker. This appeared an effective way of learning, sharing knowledge, and developing team bonds, which seemed especially useful to trainees newer to SRs. We will suggest this as a method of working when introducing the escape room in future.

### Individual differences and contextualisation

A further point of reflection comes from a participant mentioning their neurodivergence. In future, we will add additional time to escape, which should reduce the pressure on trainees. We will also provide information on the orientation in advance so that trainees know what to expect. However, it is also important to note that active learning interventions should not be considered as a one-off event. Across our DClinPsy teaching, we provide a range of self-directed learning, and individual, small, and large group active learning activities that offer trainees a framework to develop and reflect upon their learning. This range of activities and learning materials help account for individual differences.

Relatedly, we find that contextualising the escape room questions and answers at the end of the activity allows trainees to review and reflect upon their learning. One person commented that it was, “Really helpful generally to see the mistakes people tend to make, and have Paul talk us through it afterwards”. This point supports our decision to add questions on SR guidelines and discuss methodological issues rather than focus solely on search skills. We have found in the past that issues we help resolve are caused by a lack of methodological knowledge or poor practices learnt from abridged SRs undertaken in undergraduate or taught post-graduate study. One trainee summarised this by saying, “I think it highlighted some elements of a systematic review I had skimmed or even avoided when doing one before.”

Despite trainees feeling they developed their understanding of different search techniques, and mean scores for confidence in utilising search techniques ranging between ‘neutral' to ‘slightly confident', some trainees were still ‘slightly unsure' or ‘very unsure' in these skills (table 1), especially with proximity syntax. This is not surprising given that this is our first orientation with the trainees, and confidence can potentially be attributed to prior experience with SRs instead of, or in addition to, the intervention. The escape room has, however, helped us introduce these concepts at an earlier stage with this cohort, and the responses will help us scaffold future teaching and develop support resources.

Our escape room took three days to develop. This timescale is relatively short compared to creating didactic teaching materials and can therefore be a useful starting point for librarians looking to develop active learning techniques.

## CONCLUSIONS

We have demonstrated that active learning can be challenging to implement effectively, and structured guidance needs to be provided to participants ahead of and during teaching. By evaluating and reflecting on our escape room, we have illustrated how these challenges can be minimised in future.

Our SR escape room does, however, demonstrate that key methodological concepts and search skills can be taught in a fun and engaging manner, and help scaffold learning for latter in-depth teaching. As described in the appendix, the questions we chose were based on our unpublished research and the SR thesis requirement. We believe the escape room questions could equally be adapted to other forms of knowledge synthesis. Our future research will focus on a pre- and post-test to assess whether confidence to undertake SRs improves after active learning.

We would encourage librarians looking to develop innovative teaching methods to learn from our findings and utilise escape rooms as part of a suite of active learning interventions.

## Data Availability

All data supporting this study are available at https://osf.io/jwf6t/.
