# Supplementary material for: Evaluation of an online systematic review escape room for early career clinicians and doctoral students
Source: J Med Libr Assoc. 2025 Oct 23;113(4):342–8. doi: 10.5195/jmla.2025.2167 (PMC12604065; doi:10.5195/jmla.2025.2167)
Supplement: Supplementary file 1 — Appendix A [file jmla-113-4-342-s01.docx]

## Appendix A : Escape room navigation


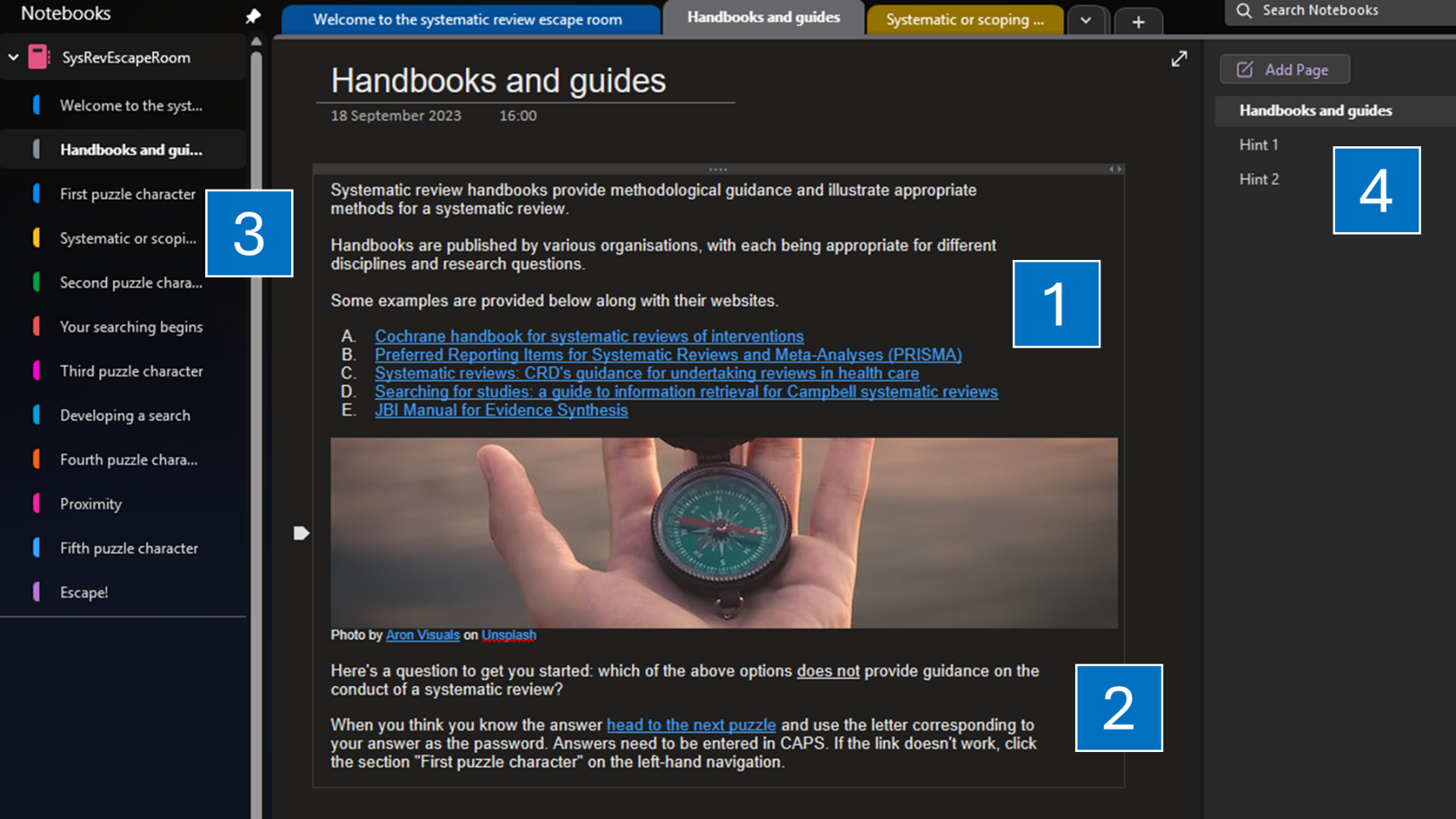


Figure A1. Question 1 of the escape room: Handbooks and guides

The context to the question and resources to explore are presented at the beginning of the puzzle (1). The question and primary means of navigating the escape room are from the hyperlinks embedded to the password protected, “puzzle character”, pages (2). Should players prefer or should they encounter an error with the navigation (which occasionally happened, and we were not able to resolve), they can navigate through the escape room using the left-hand navigation (3). Hints are provided for some questions should players require them (4).

## Escape room question development

There are five different puzzles in the ER. Our ER begins with a general introduction to the gameplay and ER navigation. The first question introduces methodological handbooks and reporting documentation. This question was chosen because trainees often conflate PRISMA reporting guidelines with methodological guidance and would often ask us questions that could be answered by following a SR handbook.

Similarly, many trainees pose research questions aligned with scoping reviews rather than the SRs required for their theses; this is why question two focuses on approaches to reviews, as per Munn et al.’s guidance [1]. Often these scoping review questions look to “identify key characteristics or factors related to a concept” [1, n.p.]; this is why the answer to this question was chosen from the possible solutions, which we then extrapolate on when contextualising the answers when closing the activity.


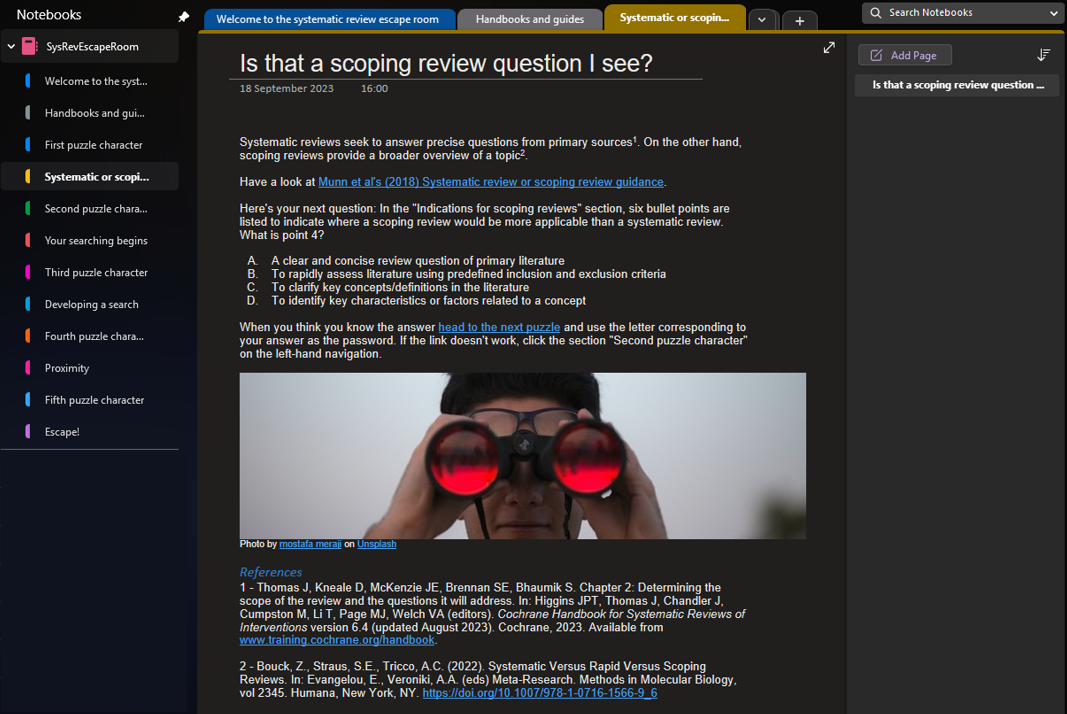


Figure A2. Question 2 of our escape room: Systematic and scoping review questions

Question three introduces Boolean logic, which is useful for trainees new to searching in a structured manner. We also introduce nesting with this question that we build upon in a latter question on proximity searching. Both are concepts that trainees have low confidence utilising correctly.


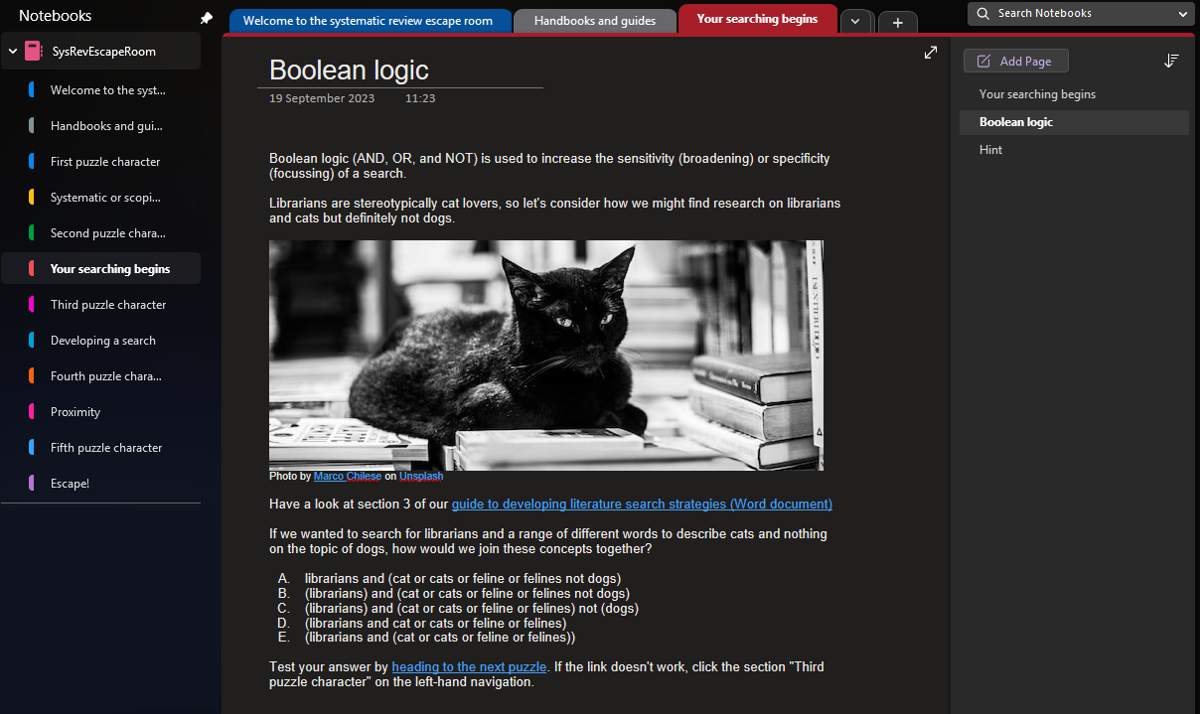


Figure A3. Page 2 of Question 3 of our escape room: Boolean logic

We then focus on search syntax by introducing truncation and wildcards in question four. Previous trainees have found wildcards a difficult concept to understand, but this is an important concept given that reviews often seek to retrieve global literature. Somewhat controversially, question four has two correct answers, which we acknowledge in the answer page. In this case, we are reinforcing the use of parenthesis that often causes issues for trainees so, for this question, one answer is *more correct* than the other!


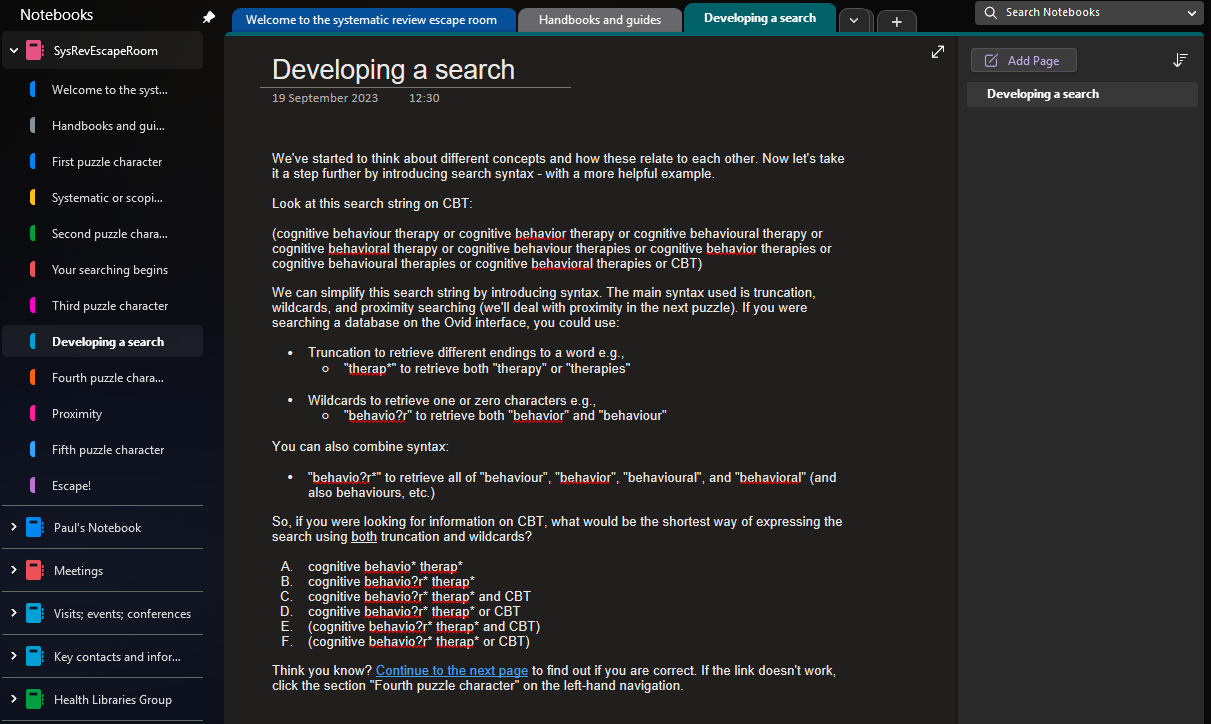


Figure A4. Question 4 of our escape room: Developing a search

Our fifth and final question concerns proximity searching that our previous, unpublished, research indicates trainees have the lowest confidence utilising.


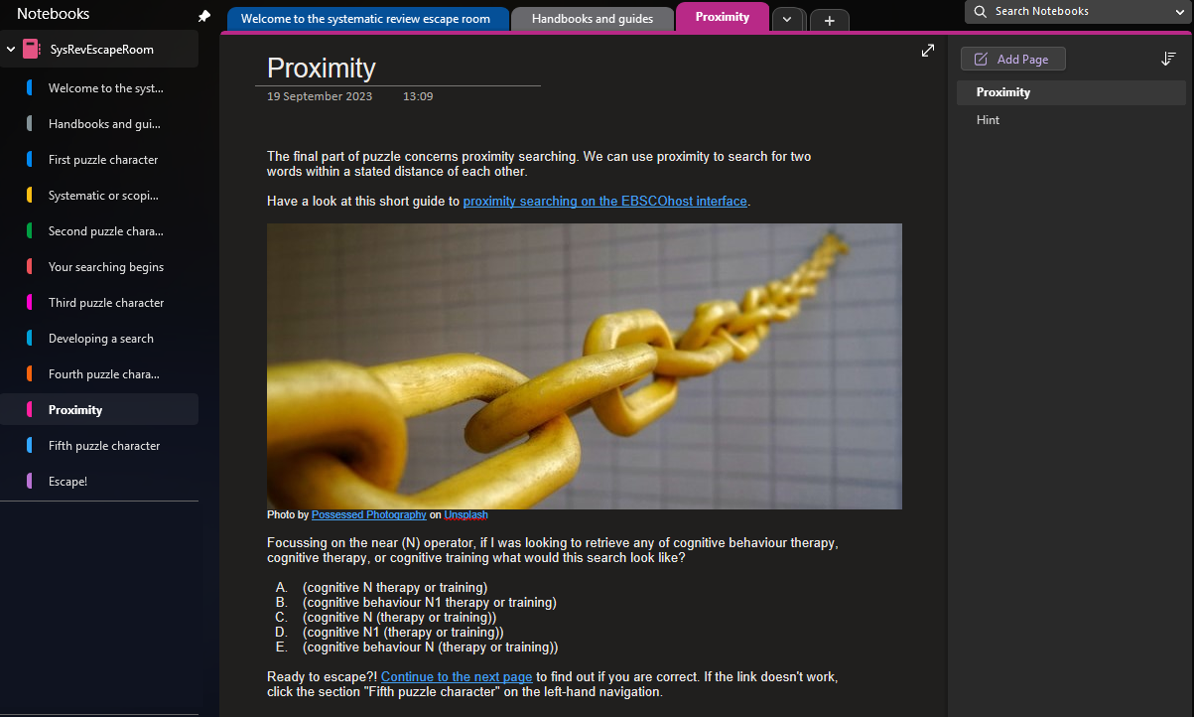


Figure A5. Question 5 of our escape room: Proximity

### References

1. Munn Z, Peters MDJ, Stern C, Tufanaru C, McArthur A, Aromataris E. Systematic review or scoping review? Guidance for authors when choosing between a systematic or scoping review approach. BMC Med Res Methodol. 2018;18(1). DOI: https://doi.org/10.1186/s12874-018-0611-x.
